# Study of Influence of Extraction Method on the Recovery Bioactive Compounds from Peel Avocado

**DOI:** 10.3390/molecules28062557

**Published:** 2023-03-11

**Authors:** Emir Martínez-Gutiérrez

**Affiliations:** CONACYT—Centro de Investigación y de Estudios Avanzados del Instituto Politécnico Nacional-Unidad Irapuato, Km. 9.6 Libramiento Norte Carretera Irapuato-León, Irapuato 36824, Guanajuato, Mexico; emir.martinez@cinvestav.mx

**Keywords:** avocado peel, extraction method, epicatechin

## Abstract

The avocado peel is a waste material from consumption avocado (*Persea americana* Mill.) with big biotechnology potential. The purpose of the present work was to study the influence of six extraction methods, maceration (M), maceration plus β-cyclodextrin (MβC), solid-state fermentation (SSF), sonication with water or ethanol, wet grinding (WG), wet grinding plus maceration (WGM), on the recovery of bioactive compounds from the avocado peel such as total phenols, epicatechin and chlorogenic acid. The results showed that the extraction method has a significant effect on the content of total phenols, the WGM method obtaining the highest value of total phenols (2143.1 mg GAE/100 g dry weight). Moreover, the results indicated that the extraction method had a significant effect on chlorogenic acid and epicatechin recovery, the WGM method obtaining the highest amount of epicatechin and chlorogenic acid, 181.7 and 244.3 mg/100 g dry matter, respectively. Additionally, the characterization of WGM extract was realized by UPLC-ESI-MS/MS and GC-MS. Thus, the WGM method allowed for obtaining good yields of recovery of phenolic compounds using an accessible technology and a more environment-friendly solvent.

## 1. Introduction

Around the world, population growth has increased the food demand, and with it organic waste has augmented, generating major environmental, economic, and social problems such as pollution of water, soil or air, waste treatment, among others [1]. Such as the case of the avocado (*Persea Americana* Mill), which is a fruit originating from Central America and Mexico that grows in tropical and subtropical regions, whose production has doubled in the last 10 years. Only in Mexico, 2,393,849 tons were produced in 2020, Mexico being the principal producer in the world [2]. In this way, avocado production generates large amounts of organic residues such as seeds and peels, which could be taken advantage as generating value-added products and at the same time contributing to the decrease in waste. Only the avocado peel represents about 16% weight of the fruit [3]. Thus, these residues (avocado peels) have been studied as a potential source of bioactive compounds or industrial interest compounds [3,4]. The bioactive compounds are defined as primary or secondary metabolites of the metabolism of plants such as phenolic compounds, among others [5]. In the case of avocado residues, the extracts obtained from the avocado peel were reported with higher contents of total phenols, flavonoids, and antioxidant capacity than the avocado seed extracts [3,6]. Moreover, epicatechin, chlorogenic acid, rutin, and quercetin were reported as compounds present in avocado peel extracts [3,4]. On the other hand, the elemental composition of the avocado peel (var. Hass) is the following: C (49.83%), N (0.97%), H (5.71%), and O (42.2%) [7].

Among applications that can have the products of the avocado peel are colorants, biopolymers, natural antioxidants, among others. However, even the information is scarce; several studies show insights about the use potential of the extract of the avocado peel with functional antioxidant properties and medicinal agents in diseases related to oxidative stress [6]. In this sense, epicatechin, a compound commonly found in the avocado peel extracts, was reported as such a compound with the possible therapeutic effect against diseases as diabetes and cancer [8]. Recently, the use of epicatechin and chlorogenic acid was observed to have an inhibitory effect on heterocyclic amines’ formation in charcoal roasted lamb meats [9]. In this way, avocado peel extract could be a product with big biotechnological potential.

However, there is little information about the strategies used for the release of bioactive compounds from the plant’s raw materials [10]. Some methods used for the release of bioactive compounds include heat reflux extraction [11]; ultrasound-assisted extraction [12]; microwave-assisted extraction [13], among others. However, most of these extraction methods use organic solvents such as methanol, ethanol, chloroform, acetone, etc., which can have negative effects on the environment and increase health risks [14]. Additionally, the modern extraction techniques are expensive methods that require specialized equipment such as microwave-assisted extraction. Meanwhile, conventional methods such as maceration, decoction, hydrodistillation, among others, are still preferred from an industrial point view [1].

In addition, there is still a long way for the development of more efficient methods that maximize the obtaining of bioactive compounds and at same time are environmentally friendly. In this sense, the extraction of polyphenols using water as a solvent, which is a food grade solvent and has a lower environmental impact compared to methanol or acetone, is an alternative that has been used with good results [3]. The addition of compounds in aqueous solutions that improve the extraction and stability of compounds through the formation of complexes has also been explored, such as the case of β-cyclodextrin, which seems to have good potential [15]. Another alternative with potential for sustainability is solid state fermentation (SSF), which has been used in the pretreatment of agro-industrial waste to improve the release and recovery of bioactive compounds [10]. SSF is a biotechnological process where the substrate serves as support for the growth of microorganisms in the absence of a free flow of water and is mainly carried out by yeasts and fungi whose advantages are low energy requirements and high product formation yields [16]. Between them, the organism utilized in SSF is *Saccharomyces cerevisiae*, which has the ability to produce alcohol from lignocellulosic biomass and tolerance to high concentrations of alcohol, among others [1]. However, reports of its application using the avocado peel as a substrate is very scarce. Moreover, it would be desirable to explore the combination of different methods in order to improve the recovery of active compounds.

Thus, the aim of this work was to evaluate the effect of method extraction on obtaining bioactive compounds (epicatechin and chlorogenic acid) from the avocado peel mainly used as a water solvent.

## 2. Results and Discussion

### 2.1. Total Phenol Content and Antioxidant Activity

The bio-compounds present in the plants can be obtained through an extraction method. However, the chosen extraction method could have an influence on the compounds obtained and at the same time have an environmental effect. The avocado peel is generally considered a residue of the consumption of avocado, but, also a source of polyphenols, which have been related with antioxidant properties [7]. The compounds that are attributed antioxidant properties generally have the capacity to free-radical scavenging, inhibition of oxidizing enzymes, among others [17]. In this sense, in the present study, the effect of the extraction method on total phenol content from the avocado peel was evaluated; the results are shown in Table 1. Previously, the humidity percentage of the avocado peel was determined, obtaining a value of 70.0% ± 0.2. Regarding the SSF method, the results only are shown at 14 days, because, in that day, the higher value was obtained (data not shown). The results showed that the extraction method had a significant effect (*p* ˂ 0.001) on the content of total phenols, the WGM method being the one which obtained the highest value of total phenols (2143.1 mg GAE/100 g dry matter), which is a combination of the WG and M methods. In fact, this value was 1.37 and 2.2 times higher than when the WG and M methods were employed separately, respectively. Thus, the combination of both methods increased the total phenol content. On the other hand, Morais et al. [18] reported 1252.31 mg GAE/100 g dry matter of the total phenol content from the dried avocado peel, using methanol as the solvent. Shi et al. [19] reported similar results (1254 mg GAE/100 g dry weight) using the Soxhlet apparatus and methanol; however, this technology was not considered environmentally friendly [20]. However, Del Castillo-Llamosas et al. [21] reported values of the total phenol content between 3130 to 4060 mg GAE/100 g dry avocado peel by means of autohydrolysis, but using a stainless-steel reactor sophisticated at high temperatures (140–180 °C). Thus, the method proposed in the present work allowed for obtaining good yields of total phenol content phenol using accessible technology and a more environment-friendly solvent.

Additionally, the antiradical activities of extracts were determined by the DPPH method; the results showed that the highest DPPH inhibition was 43.8% when the M method was used, followed by the WG method, and minor inhibition was when SSF was employed (Table 1). These results were similar to those reported by Kamaraj et al. [22], whose inhibition values were between 24.7–60.1%, depending on the extract concentration employed. However, the WGM extract showed only 65% of the inhibition observed by the M extract despite the fact that it was the extract with the highest total phenol content. This behavior in the DPPH assay could be due to the possibility that, during the chemical reaction, a steric hindrance of the extract components can occur that prevents the interaction with the DPPH, or other factors can affect the result, such as polarity, pigments, or concentration [23,24], and, therefore, to underestimate the value of the antiradical activity. So, when the concentrations of the extract components were increased, these could negatively affect the performance of the method, underestimating the value obtained.

### 2.2. HPLC Analysis and Compounds’ Identification by UPLC-ESI-MS/MS

**Figure 1:**
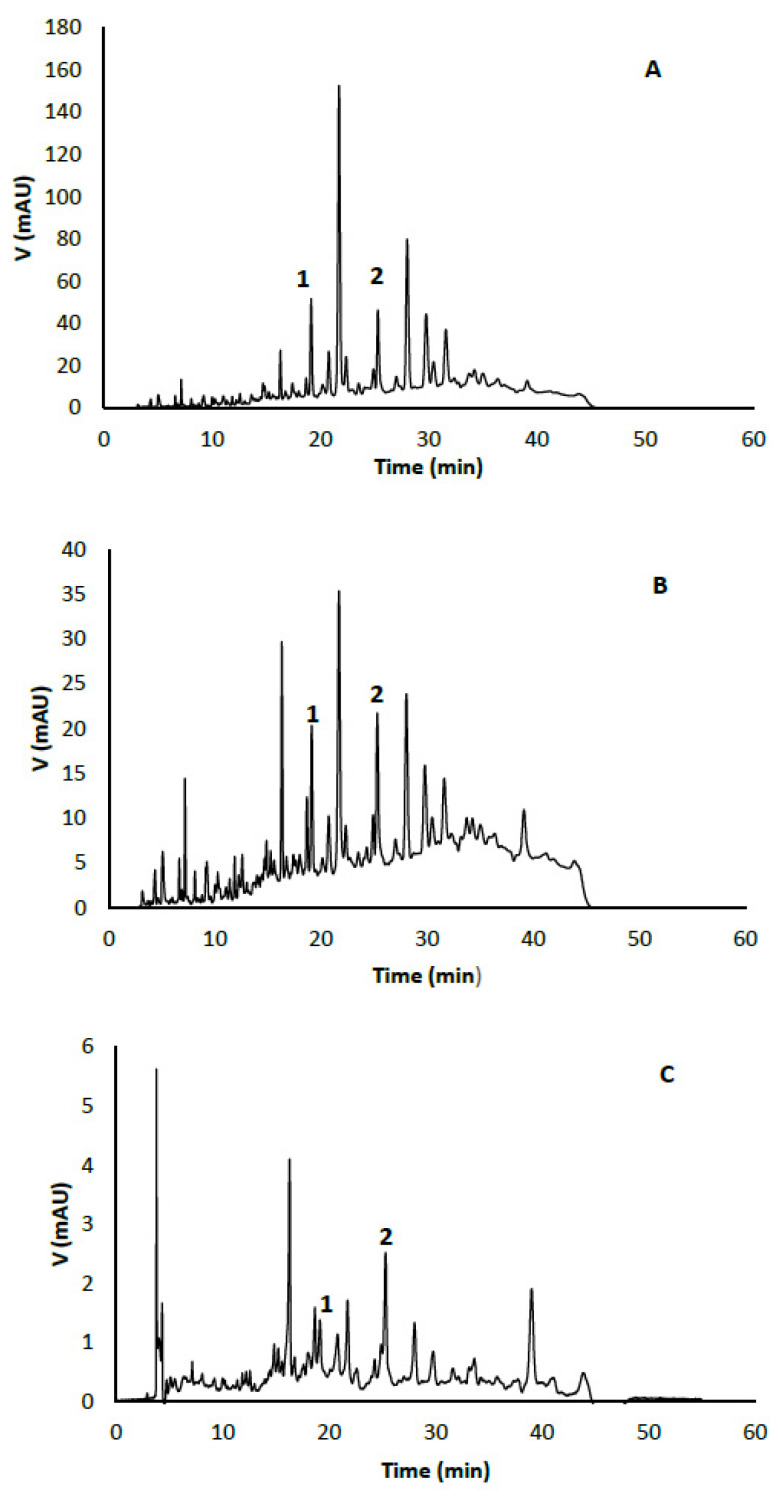
Phenolic profile of avocado peel extract obtained by (**A**) WGM method, (**B**) WG method, and (**C**) ES method at 280 nm: (1) chlorogenic acid; (2) epicatechin.

All avocado peel extracts were analyzed initially by HPLC. In this analysis, large differences were observed between the extracts as the number peaks, heights, and areas. To exemplify this, Figure 1 shows three chromatograms of avocado peel extracts (WGM, WG, and ES), chosen to have different heights (small, medium, high), noting that the WGM extract (Figure 1A) obtained higher heights in comparison with the other extracts. The same behavior was observed with the areas. Furthermore, the identification and quantification of epicatechin and chlorogenic acid were realized, on the basis of retention time, compared with authentic standards and using the calibration curves’ standard (Table 2); when the SSF method was used, these compounds were not detected in the extract. The results showed that there was a statistically significant difference (*p* ˂ 0.001) between the extraction method used and the content of chlorogenic acid obtained in the extract. The acid chlorogenic content with the smallest value was 7.2 ± 0.3 mg/100 g dry matter when the ES method was used, while the highest value was 244.3 mg/100 g dry matter when WGM was employed. Similarly, López-Cobo et al. [5] reported a concentration of chlorogenic acid of 189.89 mg/100 g dry weight in the avocado peel, but using hexane as the extraction solvent. With respect to epicatechin content, the highest value was 181.7 ± 31.4 mg/100 g dry weight when the WGM method was utilized; this value was up to eight times highest than when the WS method was used. 

Therefore, the extraction method had a significant effect (*p* ˂ 0.001) on the epicatechin concentration obtained. Figueroa et al. [25] reported a concentration of epicatechin 59/100 g peel dm using a microwave-assisted extraction (MAE) and ethanol (36%) as the solvent. In other work, 129.79 µg of epicatechin/100 g dry weight was obtained using methanol as the solvent and stirring for four hours [20]. These results indicate that it is possible to obtain a good yield using an environment-friendly solvent and an accessible method such as the WMG method, which combines the mechanical action and maceration a 70 °C.

Subsequently, the extract obtained by the WMG method was analyzed by UPLC-ESI-MS-MS because this extract presented the greatest number of peaks and the highest areas of the target compounds (epicatechin and chlorogenic acid). The phenolic compounds were identified on the basis of mass spectra, retention time, comparison with authentic standards when available, and previously reported information; the results are shown in Table 3.

Through this study, it was possible to identify 26 tentative compounds; among them were found phenolic acids such as benzoic acid, gentisic acid and vanillic acid, hydroxycinnamic acid esters (e.g., chlorogenic acid), flavonoids (e.g., catechin and epicatechin), among others. Some of these compounds have been related to different promising properties, such as the case of the hydroxybenzoic acids such as salicylic acid and gentisic acid, which were related with an effect that was pharmacologically active, such as decreasing oxidative stress and inflammation [32], while the quinic acid was related with properties such as antioxidant, antidiabetic, analgesic, among others [33]. Moreover, chlorogenic acid (3-caffeoylquinic acid) was identified; this compound has been related with roles such as antioxidant activity, cardioprotective activity, neuroprotective activity, anti-obesity, among others [34]. Between flavonoids, epicatechin was identified and was one of the most abundant compounds in the extract. Epicatechin is a secondary metabolite that shows antioxidant and anti-inflammatory activities, prevents diabetes, protects the nervous system, among others [35], whereas naringin presents anti-inflammatory and anticancer activities [36]. Thus, the extract of the avocado peel can be a source of polyphenols with a wide range of applications.

Additionally, a compound labeled as unknown 2 was detected, and it was not possible to achieve an unambiguous identification, but, according to m/z (609.28) and reports of previously works, it could be multinoside A [25], quercetin-rhamnoside-hexoside [30] or kaempferol-dihexosa [23]; the rutin was discarded because the retention time was not the same as the authentical standard.

### 2.3. GC-MS Analysis

An analytical characterization of compounds present in avocado peel extract (WGM) was carried out using GC-MS. The identification was realized on the basis of the retention time value and fragmentation patterns of the mass spectra. GC-MS results of the extract confirmed the presence of 38 compounds that were tentatively identified and others whose unambiguous identification was not possible (Table 4). Between these compounds are sugars such as glucose and fructose and sugars’ alcohols (polyalcohols) such as glycerol, pentitol, and glucitol, which could be used as sweeteners [37]. Moreover, malic acid was identified, which presented one of the highest peaks. Malic acid is a compound predominant in several fruits, and among their applications are their use as an acidulant and taste enhancer in several food products [38]. Too, epicatechin was identified, presenting one of the highest peaks, which, as mentioned before, presents antioxidant activity. Moreover, fatty acids such as octanoic and palmitic acid were identified. 

### 2.4. Comparison of the Extraction Methods

In the present work, six extraction methods for the recovery of bioactive compounds from the avocado peel were evaluated, focusing mainly on the recovery of epicatechin and chlorogenic acid and using water as the solvent, except for the sonication method in which ethanol was also evaluated. These methods included conventional methods such as maceration and more advanced technologies such as sonication. The latter is considered a “Green Food Processing” technology, whose focus is simplicity, energy efficiency, and economy [39]. The results indicated that, with the WGM method, it was possible to recover a greater amount of both chlorogenic acid and epicatechin, though, in the case of chlorogenic, a significative difference was not observed when WG and WGM were employed. However, in comparison with the other methods, the difference was bigger, for example, comparing the MGW with the WS method, when WGM was employed up to 9 and 8 times more chlorogenic acid and epicatechin were obtained, respectively. On the other hand, the addition of β-cyclodextrin did not improve the extraction, while, when the SSF method was used, it was not possible to detect chlorogenic acid or epicatechin under the evaluated conditions, so it would be desirable to explore other conditions in order to obtain better results. The WGM method combines two methods, wet grinding and maceration; both are conventional technologies that together managed to enhance the recovery of bioactive compounds. This indicates that conventional technologies still have great potential in the recovery of bioactive compounds and may even have better performance than new technologies, despite the fact that it was indicated that these technologies are not sufficient to extract the maximum number of materials [39], while new technologies were attributed an improvement in mass transfer, cell permeability, and high extraction yields [40]. Therefore, the effectiveness of the extraction depends on a large number of factors, including type of method, temperature, solvent, time, use of one or more methods, etc. Therefore, it is necessary to continue exploring different conditions and combinations of technologies in order to maximize the recovery of bioactive compounds.

## 3. Materials and Methods

### 3.1. Chemical and Reagents

Folin-Ciocalteu’s phenol reagent, β-cyclodextrin, catechin, chlorogenic acid, (−)-epicatechin, gallic acid, and DDPH (2,2-diphenyl-1-picrylhydrazyl) were purchased from Sigma-Aldrich. All reagents used as standards in the UPLC-ESI-MS/MS analysis were HPLC grade (Sigma-Aldrich). Acetonitrile was HPLC grade, and all others reagents such as ethanol were ACS reagent grade and were purchased from J.T. Baker.

### 3.2. Microorganism

*S. cerevisiae* (strain S288C) was selected for solid-state fermentation and was maintained on yeast potato dextrose agar (YPD) Petri plates at 4 °C. The yeast colonies were revived by transferring onto fresh liquid YPD medium and incubated at 28 °C before use.

### 3.3. Preparation of Feed Stock and Extraction Methods

The avocado peel (var. Hass) was collected from markets, coffee shops, and homes. The avocado peels were rinsed and collocated on absorbent paper to remove the water excess and were cut in pieces of approximately 1 to 2 cm^2^. Subsequently, for the first four methods, the biomass was ground in a commercial food processor and sieved using a sieve of 2000 µm. The resultant avocado peel was placed in bags and stored in refrigeration (−20 °C). Moreover, the humidity content was carried out by weight difference; the sample was dried at 60 °C over 24 h.

Various extraction methods were evaluated, which are described as a continuation: (a) 40 g of avocado peel plus 200 mL of distillated water were maintained at 70 °C with agitation over 1 h (called maceration (M)); (b) in the second method (called MβC), the anterior process with the addition of β-cyclodextrin (1% *w*/*v*) was used; (c) in the third method, the sonication was evaluated using water or ethanol as the solvent (called WS and ES, respectively); in this method, 1 g of the substrate and 10 mL of the solvent were mixed for 1 min in a vortex, and were subsequently sonicated over 15 min (frequency 40 KHz, power 150 W, mrc Ultrasonic Cleaner D150); (d) in the fourth method, the solid-state fermentation (SSF) was evaluated, which later is described (called SSF); (e) in the fifth method, after cutting the avocado peel to 1 or 2 cm^2^, this biomass (40 g), plus 200 mL of water, was ground using a commercial blender (FARBERWARE, 120 V, 60 Hz, 900 W) over 30 s (called: wet grind (WG)); (f) in the last method, the wet grind method plus a last maceration stage with agitation at 70 °C during 1 h was used (called WGM). All extracts were filtered, protected from the light, and stored at −20 °C.

#### Solid-State Fermentation (SSF)

For SSF, 250 mL Erlenmeyer flasks containing 40 g avocado peel were autoclaved at 121 °C for 15 min and after were cooled. The flasks were inoculated with 2.5 × 10^7^ cell/g. After being gently mixed, the fermentations were conducted for 28 days at 28 °C. The experiments were realized by triplicate. A total of 1 g of sample was removed after 7, 14, 21, and 28 days and stored at −20 °C and protected from light. For extraction, 1 g of the sample was added to 10 mL of distillated water and shaken in vortex for 1 min. After, the sample was maintained in a water bath at 70 °C for 1 h, and finally these last two steps were repeated. The extracts were filtrated and stored at −20 °C.

### 3.4. Total Phenolic Content

The total phenolic content was determined for the Folin-Ciocalteu method [41] with modifications: 100 µL of extract were mixed with 0.5 mL Folin-Ciocalteu reactive (0.2 N) and 0.4 mL of Na_2_CO_3_ (7.5%). The mixture was incubated at room temperature in the dark over 30 min; the absorbance was measured at 760 nm. The total phenolic content was calculated through a standard curve of gallic acid; the result was expressed as gallic acid equivalents/100 mg dry matter.

### 3.5. Antioxidant Activity

Radical scavenging activity was determined according to Brand-Williams et al. [42] using DPPH (1-1-diphenyl-2-picrilhdrazyl). A total of 30 µL of the sample plus 2 mL of DPPH solution were shaken in vortex and after kept at room temperature in the dark for 30 min. Absorbance was measured at 515 nm. Radical scavenging activity was expressed as the inhibition percentage and was determined using the following equation:

% inhibition = ((Absorbance of blank − Absorbance of sample)/(Absorbance of blank)) × 100(1)

### 3.6. HPLC Analysis

The determination and separation of compounds were realized by HPLC [43,44]. Previously, the samples were filtrated in the nylon membrane (0.45 µm). A total of 10 µL of the sample was automatically injected into an Agilent 1200 series (Agilent Technologies, Waldbronn, Germany ) equipped with a UV-Vis detector with a diode array. A column YMC-ODS-AM C-18 (250 × 4.6 mm) was utilized, and the mobile phase was composed by acetic acid (1%) in water (phase A) and (100%) acetonitrile (phase B). The gradient elution was the following: 0 min, 0% B; 5 min, 5% B; 10 min, 10% B; 30 min 15% B; 40 min, 15% B; 42 min, 0% B. The flow was 1 mL/min with λ = 280 nm at 25 °C. Epicatechin and acid chlorogenic were identified by taking the retention time of the standards, and their concentration was determined by interpolation of the peaks’ area in the standard calibration curve.

### 3.7. UPLC-ESI-MS/MS Analysis

The analysis of the WGM extract previously lyophilized was realized using an Ultra-High-Performance-Liquid Chromatography (Waters Corp., Milford, MA, USA) coupled to an Electrospray Ionization-Tandem Mass Spectrometry, Triple Quadrupole (Xevo, TQS, Waters Corp., Wexford, Ireland). The injection volume was 1 µL, and a column Acquity BEH C18 (50 mm ± 1.7 × 2.1 µm) was used for the separation. The temperatures of the column and the sample were 35 °C and 6 °C, respectively. The mobile phase was composed by formic acid (7.5 mM) in water (phase A) and 100% acetonitrile (phase B). The gradient elution was as follows: 0 min 3% B, 1.23 min 9% B, 3.82 min 16% B, 11.4 min 50% B, 13.24 min 3% B, 15 min 3% B.

The ionization of the sample was by electrospray, capillary voltage 2.25 kV, cone voltage 30 V, source temperature 150 °C, desolvation temperature 400 °C, cone gas rate 150 L/h, collision gas flow (0.15 mL/min), MS mode 5, MS/MS mode 20.

### 3.8. GC-MS Analysis

The WGM sample was derivatized before GC-MS analysis. The sample derivatization was carried out as follows: about 2 mg of the lyophilized sample were weighed out and dissolved with 80 µL of methoxyamine hydrochloride (20 mg/mL in pyridine) and incubated for 90 min at 37 °C. Subsequently, 80 µL of N-methyltrifluoroacetamide (MBSTFA) plus 1% trimethylchlorosilane (TMCS) were added and incubated for 30 min at 37 °C. One microliter of the sample was injected into the GC/MS system. The GC/MS system consisted of the gas chromatography coupled to mass spectrometry (Agilent 5977A/7890B GC–MS, Santa Clara, CA, USA) with an automatic autosampler (G4513A, Agilent). The HP5ms column (30 m × 250 µm × 0.25 µm) of the Agilent brand was used to carry out the separation of the analytes using helium (99.9999% pure) as the mobile phase. The equipment was calibrated with the Agilent brand perfluorotributylamine (PFTBA) standard. An untargeted analysis was performed with an acquisition range of 50 to 600 Da with an ionization energy of 70 eV. A scan rate of 3 scan/s with a 20 Hz digital scan was used. The specified parameters of the run were the following: inlet temperature 200 °C, quadrupole temperature 150 °C, source temperature 250 °C, flow rate 1 mL/min, standard injection, injection volume 1 µL, split (splitsses). The running method consisted of a ramp of 60 °C to 325 °C with increases of 10 °C/min and 37 min of run time. All reagents and solvents used were pure analytical grade.

The spectral deconvolution and peak alignment were realized with the Mzmine2 software, and the identifications were realized using the National Institute of Standards and Technology (NIST) 2.0 spectral library. It was considered as a correct (putatively) identification when the match was higher than 70% (R > 70%); values below this limit were marked as unknown or omitted.

### 3.9. Statistical Analysis

One-way analysis, ANOVA, and Tukey’s were evaluated using SPSS. The *p* values ˂ 0.05 were considered as significant differences.

## 4. Conclusions

In this study, bioactive compounds were obtained from the avocado peel by different extraction methods. The influence of the extraction method was evaluated by using total phenol content and epicatechin and chlorogenic acid content, where the method with the highest recovery of bioactive compounds was MGW. The MGW method combines two conventional techniques that together potentiated the recovery of bioactive compounds, being an attractive and accessible technology with potential applications both on a small and large scale.

## Figures and Tables

**Table 1:** Total phenol content and antiradical activity in the avocado peel extract obtained by different extraction methods.

Extraction Method	mg GAE ^1^/100 g Dry Matter	% DPPH Inhibition
M	972.9 ± 118.6 ^b^	43.8 ± 3.1 ^c^
MβC	845.4 ± 113.2 ^a,b^	29.2 ± 4.0 ^b^
WS	635.9 ± 19.7 ^a^	25.7 ± 1.0 ^b^
ES	953.8 ± 85.6 ^b^	26.1 ± 0.4 ^b^
SSF (14 d)	800.2 ± 72.7 ^a,b^	23.7 ± 1.2 ^a,b^
SSF (control)	649.4 ± 53.5 ^a^	17.1 ± 2.3 ^a^
WG	1562.2 ± 137.9 ^c^	39.4 ± 1.5 ^c^
WGM	2143.1 ± 85.9 ^d^	28.5 ± 4.6 ^b^

^1^ mg gallic acid equivalents (GAE). Values are the mean of three measurements ± standard deviation; different letters mean there are significant differences (*p* ˂ 0.001).

**Table 2:** Chlorogenic acid and epicatechin content in the avocado peel extract obtained by different extraction methods.

Title ^1^	Chlorogenic Acid (mg/100 g Dry Matter)	Epicatechin (mg/100 g Dry Matter)
M	73.48 ± 19.14 ^c^	43.83 ± 6.10 ^a^
MβC	53.60 ± 4.95 ^b,c^	45.84 ± 13.81 ^a^
WS	29.61 ± 5.25 ^a,b^	22.85 ± 1.52 ^a^
ES	7.22 ± 0.30 ^a^	24.84 ± 0.66 ^a^
WG	241.88 ± 7.42 ^d^	133.64 ± 8.35 ^b^
WGM	244.33 ± 24.44 ^d^	181.71 ± 31.79 ^c^
SFF	N.D. *	N.D. ^a^*

^1^ Values are the mean of three measurements ± standard deviation; different letters mean there are significant differences (*p* ˂ 0.001). * Not detected. Epicatechin curve: y = 6.134x − 27.292 R^2^ = 0.9998. Chlorogenic acid curve: y = 2.7015x − 5.1712 R^2^ = 0.9964.

**Table 3:** Identification of phenolic compounds present in the avocado peel extract.

No.	Proposed Compound	RT (min)	m/z	Fragmentations	Molecular Formula	Reference
1	Quinic acid *	0.61	191.2	85.06	C_7_H_12_O_6_	[26]
2	shikimic acid *	0.66	173.18	111.07	C_7_H_10_O_5_	[27]
3	Gentisic acid *	3.44	153.15	108.2	C_7_H_6_O_4_	[26]
4	4-hydroxybenzoic acid *	3.51	137.04	93.05	C_7_H_6_O_3_	[26]
5	Chlorogenic acid *	3.80	353.1	191.2	C_16_H_18_O_9_	[28]
6	4-O-caffeoylquinic acid	4.65	353.3	179.06	C_16_H_18_O_9_	[26]
7	Vanillic acid *	4.13	167.18	152.02		[26]
8	Caffeic acid *	4.27	179.19	135.08	C_9_H_8_O_4_	[26]
9	Syringic acid *	4.45	197.21	182.5	C_9_H_10_O_5_	[26]
10	Coumaric acid *	5.45	163.24	119.09	C_9_H_8_O_3_	[26]
11	Ellagic acid *	6.31	301	229	C_14_H_6_O_8_	
12	Ferulic acid	5.99	193.24	134.04	C_10_H_10_O_4_	[23]
13	Sinapic acid *	6.04	223.24	164.06	C_11_H_12_O_5_	[28]
14	Benzoic acid *	6.79	121.1	77.1	C_7_H_6_O_2_	[26]
15	trans-cinnamic acid *	8.83	147.17	103.08	C_9_H_8_O_2_	
16	2-hydrobenzoic acid *	6.78	137.04	93.05	C_7_H_6_O_3_	[29]
17	Diccaffeoylquinic acid	7.19	515.45	353.2	C_25_H_24_O_12_	[7]
18	Coumaric acid (isomer)	4.0	163.24	119.08	C_9_H_8_O_3_	
19	Procyanidin B1 *	3.45	577.44	289.18	C_30_H_26_O_12_	[26]
20	Catechin *	3.88	289.164	245.2	C_15_H_14_O_6_	[26]
21	Procyanidin B2 *	4.39	577.44	289.18	C_30_H_26_O_12_	[26]
22	Epicatechin *	4.79	289.164	245.2	C_15_H_14_O_6_	[30]
23	Quercetin glucoronide	6.27	477.26	301.1	C_21_H_20_O_12_	[30]
24	Quercetin 3-O-glucoside	6.24	463.36	300.42	C_21_H_20_O_12_	[31]
25	Naringin *	6.94	579.32	151.02	C_27_H_32_O_14_	[28]
26	Eriodictyol	8.33	287.28	151.04	C_15_H_12_O_6_	
27	Unknown 2	6.61	609.28	300.24	C_27_H_30_O_16_	

* Compounds identified by comparison with an authentical standard.

**Table 4:** Identification of compounds present at the avocado peel by GC-MS.

No.	TR	Molecular Formula	Molecular Weight (g/mol)	Proposed Compound
1	6.448	C_3_H_6_O_3_	90.08	Lactic acid
2	6.634	C_2_H_4_O_3_	75.06	Glycolic acid
3	7.019	C_3_H_7_NO_2_	89.09	Alanine
4	8.424	C_3_H_4_O_4_	104.0615	Malonic acid
5	8.615	C_5_H_11_NO_2_	117.15	Valine
6	8.992	C_7_H_6_O_2_	122.12	Benzoic acid
7	9.186	C_8_H_16_O_2_	144.21	Octanoic acid
8	9.434	H_3_PO_4_	98.0	Phosphoric acid
9	9.437	C_3_H_8_O_3_	92.09	Glycerol
10	9.723	C_4_H_9_NO_2_	103.12	4-Aminobutanoic acid
11	9.903	C_4_H_6_O_4_	118.09	Succinic acid
12	10.217	C_3_H_6_O_4_	106.08	Glyceric acid
13	10.331	C_4_H_4_O_4_	116.1	Maleic acid
14	10.434	C_5_H_6_O_4_	130.09	Methylmaleic acid
15	10.611	C_3_H_7_NO_3_	105.09	Serine
16	10.974	C_4_H_9_NO_3_	119.1192	Threonine
17	12.262	C_4_H_6_O_5_	134.0874	Malic acid
18	12.645	C_4_H_7_NO_4_	133.11	Aspartic acid
19	12.731	C_4_H_9_NO_2_	103.12	4-Aminobutanoic acid
20	12.977	C_5_H_12_O_5_	152.15	Pentitol
21	13.22	C_7_H_6_O_5_	170.12	2,3,4-Trihydroxybutiric acid
22	13.294	C_5_H_6_O_5_	146.11	2-Oxoglutaric acid
23	13.815	C_5_H_9_NO_4_	147.13	Glutamic acid
24	13.895	C_9_H_11_NO_2_	165.19	Phenylalanine
25	15.533	C_5_H_10_N_2_O_3_	146.14	Glutamine
26	15.96	C_7_H_10_O_5_	174.15	Shikimic acid
27	16.115	C_6_H_8_O_7_	192.024	Citric acid
28	16.772	C_6_H_12_O_6_	180.16	Fructose
29	17.043	C_6_H_12_O_6_	180.16	Glucose
30	17.224	C_6_H_12_O_6_	180.16	Glucose isomer
31	17.27	C_9_H_11_NO_3_	181.19	Tyrosine
32	17.442	C_6_H_14_O_6_	180.17	Glucitol
33	18.031	C_6_H_12_O_7_	196.16	Gluconic acid
34	18.146	C_16_H_32_O_2_	256.4	Palmitic acid
35	18.89	C_6_H_12_O_6_	180.16	Myo-inositol
36	23.572	C_12_H_22_O_11_	342.3	Sucrose
37	24.953	C_15_H_14_O_6_	290.26	Epicatechin ^1^
38	25.091	C_15_H_14_O_6_	290.26	Catechin isomer

^1^ The identification of epicatechin was compared with its standard.

## Data Availability

Data are available upon request from the corresponding author.

## Sample Availability

Samples of the compounds are not available from the authors.
